# Comparison of different cell type correction methods for genome-scale epigenetics studies

**DOI:** 10.1186/s12859-017-1611-2

**Published:** 2017-04-14

**Authors:** Akhilesh Kaushal, Hongmei Zhang, Wilfried J. J. Karmaus, Meredith Ray, Mylin A. Torres, Alicia K. Smith, Shu-Li Wang

**Affiliations:** 1 Division of Epidemiology, Biostatistics, and Environmental Health, University of Memphis, Memphis, 38152 TN USA; 2 Winship Cancer Institute, Emory University, 1365 Clifton Rd. NE, Atlanta, 30322 GA USA; 3 Department of Radiation Oncology, Emory University School of Medicine, 1365 Clifton Rd. NE, Atlanta, 30322 GA USA; 4 Department of Psychiatry and Behavioral Sciences, Emory University School of Medicine, 101 Woodruff Circle, Suite 4000, Atlanta, 30322 GA USA; 5 National Institute of Environmental Health Sciences, National Health Research Institutes, Miaoli, Taiwan

**Keywords:** Cell-type composition, CpG sites, Genome-scale DNA methylation, Surrogate variables

## Abstract

**Background:**

Whole blood is frequently utilized in genome-wide association studies of DNA methylation patterns in relation to environmental exposures or clinical outcomes. These associations can be confounded by cellular heterogeneity. Algorithms have been developed to measure or adjust for this heterogeneity, and some have been compared in the literature. However, with new methods available, it is unknown whether the findings will be consistent, if not which method(s) perform better.

**Results:**

*Methods*: We compared eight cell-type correction methods including the method in the minfi R package, the method by Houseman et al., the Removing unwanted variation (RUV) approach, the methods in FaST-LMM-EWASher, ReFACTor, RefFreeEWAS, and RefFreeCellMix R programs, along with one approach utilizing surrogate variables (SVAs). We first evaluated the association of DNA methylation at each CpG across the whole genome with prenatal arsenic exposure levels and with cancer status, adjusted for estimated cell-type information obtained from different methods. We then compared CpGs showing statistical significance from different approaches. For the methods implemented in minfi and proposed by Houseman et al., we utilized homogeneous data with composition of some blood cells available and compared them with the estimated cell compositions. Finally, for methods not explicitly estimating cell compositions, we evaluated their performance using simulated DNA methylation data with a set of latent variables representing “cell types”.

*Results*: Results from the SVA-based method overall showed the highest agreement with all other methods except for FaST-LMM-EWASher. Using homogeneous data, minfi provided better estimations on cell types compared to the originally proposed method by Houseman et al. Further simulation studies on methods free of reference data revealed that SVA provided good sensitivities and specificities, RefFreeCellMix in general produced high sensitivities but specificities tended to be low when confounding is present, and FaST-LMM-EWASher gave the lowest sensitivity but highest specificity.

**Conclusions:**

Results from real data and simulations indicated that SVA is recommended when the focus is on the identification of informative CpGs. When appropriate reference data are available, the method implemented in the minfi package is recommended. However, if no such reference data are available or if the focus is not on estimating cell proportions, the SVA method is suggested.

**Electronic supplementary material:**

The online version of this article (doi:10.1186/s12859-017-1611-2) contains supplementary material, which is available to authorized users.

## Background

Whole blood is frequently utilized in genome-wide association studies of DNA methylation patterns in relation to environmental exposures or clinical outcomes. However, for DNA methylation assessed from whole blood, the association between DNA methylation and an exposure of interest could be confounded by cellular heterogeneity [1, 2]. In larger epidemiological studies, it is not feasible to isolate and profile every individual cell subset. Thus, several algorithms have been developed to measure and adjust for cellular heterogeneity in whole blood.

Houseman et al. proposed a method to infer the cell mixture proportions based on a regression calibration technique, which uses an external validation dataset to calibrate the model and correct for the bias [3]. Jaffe and Irizarry [4] modified the Houseman et al.’s algorithm and tailored it to predict cell mixture composition of DNA-methylation profiles obtained from a different Illumina platform. This cell type correction method is implemented in Bioconductor [5] package minfi [6]. The above two approaches require external validation datasets and are designed to identify cell mixtures in tissues such as whole blood.

Apart from these two reference-based techniques, non-reference-based methods have also been developed. An advantage of these non-reference-based methods is that they can be applied to other tissues in addition to blood. Zou et al. developed a non-reference-based method, FaST-LMM-EWASher. This approach is built upon linear mixed models with top principal components as the covariates. Another set of methods infer latent variables for cell type compositions, which are then included in association assessments. These methods include RefFreeEWAS and its recently improved version (RefFreeCellMix), surrogate variable analysis (SVA), and ReFACTor [7–10]. RefFreeEWAS [7] and RefFreeCellMix [8] both utilize singular value decompositions (SVDs) and extract latent subject and cell-specific effects, but RefFreeCellMix incorporated additional constraints and utilities aiming to reduce the occurrence of false positives. Surrogate variable analysis (SVA) [9], based on SVDs of residuals in linear regressions, uses permutations to identify statistically significant eigen-vectors and consequently infer potential confounding factors (surrogate variables). A Bioconductor package is available to estimate surrogate variables using this approach [9]. Finally, ReFACTor [10] is based on principal component analyses on a set of potentially informative CpG sites.

Removing unwanted variation (RUV) is an approach different from the aforementioned methods and it was designed to estimate cell type heterogeneity and built upon factor analyses. This approach utilizes reference CpGs inferred from a reference database, based on which factor analyses are conducted. The factors are then included in subsequent analyses for the purpose of adjusting for cell type effects. Although a reference database is needed, this method does not estimate cell type proportions as done in the minfi package and in the Houseman et al. method.

In our earlier work Kaushal et al. [11], we compared five methods, Houseman et al., minfi, FaST-LMM-EWASher, RefFreeEWAS, and a method by use of SVA. McGregor et al. [12] compared the methods noted above except for ReFACTor and RefFreeCellMix and focused on assessment of associations between DNA methylation and a variable of interest by taking cell type compositions into account. With the additional methods included (ReFACTor and RefFreeCellMix), it was unclear whether the findings would be consistent with those in McGregor et al., Kaushal et al., and if not, which method(s) might perform better. To this end, we first applied each cell type correction method (Houseman et al., minfi, RUV, FaST-LMM-EWASher, ReFACTor, RefFreeEWAS, and RefFreeCellMix) as well as the surrogate variable analyses (SVA) to two real data sets. For these real data sets, we evaluated the association between genome-scale DNA methylation and a variable of interest adjusting for cell type compositions. We assessed the agreement within each data set in terms of identified CpGs between different methods and the consistency of findings of each method between different data sets. We also qualitatively compared different approaches based on existing knowledge about the sparsity of informative genes, pathways and genetic functions. For the method implemented in the minfi package and that proposed by Houseman et al., we utilized a homogeneous real data set with some blood cells composition available and compared the true cell counts with the estimated cell compositions. For methods free of reference groups (FaST-LMM-EWASher, RefFreeEWAS, RefFreeCellMix, ReFACTor, and SVA), we further utilized simulated data generated under different scenarios to compare different methods, which, combined with findings from the real data, enabled us to comprehensively assess each method.

## Results

### Findings from prenatal arsenic exposure and DNA methylation data

We used genome-scale DNA methylation data from a birth cohort study consisting of 64 cord blood samples examining multiple prenatal factors in relation to child health outcomes, pilot of the nationwide Taiwan Maternal and Infant Cohort Study [13, 14].

**Fig. 1:**
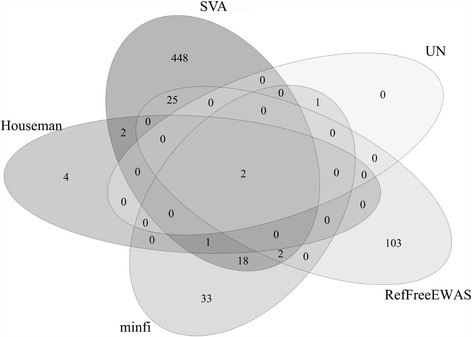
Venn diagram illustrating the overlap of identified CpG sites that were associated with prenatal arsenic exposure at FDR level of 0.05 after incorporating estimated cell type compositions by different methods for the association study of prenatal arsenic exposure with DNA-methylation. Results from Houseman et al., *minfi*, *RefFreeEWAS*, and *SVA* as well as the analyses without adjusting for cell types are displayed (Results from other methods are in the text). “*UN*”: results from an analysis without adjusting for cell type compositions

Via linear regressions, we assessed the association of DNA methylation at each CpG site across the whole genome with prenatal urinary arsenic exposure levels (a continuous measure), adjusting for cell-type effects with cell type information inferred from one of the eight methods. For each method, we recorded the number of CpGs showing statistically significant associations with prenatal urinary arsenic exposure after adjusting for multiple testing by controlling false discovery rate (FDR) at 0.05. ReFACTor identified the largest number of CpGs (~60,000) and no CpGs were detected by FaST-LMM-EWASher (Table 1). RefFreeCellMix also identified a large number of CpGs (~3000). SVA and RefFreeEWAS detected more CpGs compared to the remaining methods. (Table 1). Next, we assessed the number of identified CpGs that overlapped between different methods. The diagram in Fig. 1 shows the overlap of CpG sites from four approaches (Houseman et al., minfi, RefFreeEWAS, and SVA) as well as the analyses without adjusting for cell types (we did not include all eight methods in this Venn diagram for clarity). Results from SVA showed the best agreement with findings from the other four analyses (Fig. 1). Two identified CpG sites cg06434480 and cg10662395 were common to all these five analytical methods labeled in Fig. 1. Further comparisons indicated that CpG site cg10662395 was also identified by RefFreeCellMix and RUV, and was the only CpG site to overlap among all seven analyses (Houseman et al., minfi, RefFreeEWAS, SVA, RefFreeCellMix and RUV, as well as the analyses without adjusting for cell types). Although ReFACTor identified the largest number of CpGs, they did not overlap with the joint findings from the aforementioned seven analyses. Overall, CpGs identified via SVA overlapped with those from the Houseman et al. method, minfi and RefFreeEWAS (*p*-value < 0.0001, Table 1, Fig. 1. The definition of percentage overlap is given in the Methods section). One of the two CpGs (cg06434480 and cg10662395), cg06434480, is located within 200 base pairs of the transcription start site of gene *HMGCR* (3-hydroxy-3-methylglutaryl-CoA reductase) which is known to be associated with inorganic arsenic exposure [15]. In a study conducted in humans, Mono-methylated arsenic (MMA) was found to downregulate the gene expression of *HMGCR*, a gene involved in cholesterol biosynthesis [16]. The other CpG, cg10662395, is located in the body region of gene *HCN2* (hyperpolarization activated cyclic nucleotide gated potassium channel 2). This gene was not found to be directly associated with arsenic exposure in the literature, but *HCN2* has been known to regulate pacemaker activity in the heart and the brain of mice and humans [17, 18]. Arsenic has been found to induce QT interval (i.e., time between initial deflection of QRS complex to the end of T wave) prolongation probably by altering potassium ion channel [19].

**Table 1:** Number of significant CpG sites with and without cell type correction and overlap with the SVA method (data on prenatal arsenic exposure and DNA methylation)

Method	Identified CpGs (*N*)^#^	Overlap with SVA (%)	*p*-value^##^
Houseman et al.	10	1.20	<0.0001
minfi	57	4.62	<0.0001
SVA	498	–	–
RefFreeEWAS	133	6.01	<0.0001
RefFreeCellMix	2932	0.60	1.0
ReFACTor	58,871	13.03	1.0
EWASher ^a^	0	0.0	–
RUV	356	0.20	1.0
Unadjusted ^b^	3	0.60	<0.0001

^#^The selection of CpG sites is based on FDR-adjusted *p*-values (FDR is controlled at 0.05)

^##^ *P*-value is based on Fishers exact test for overlap with results from SVA. The null hypothesis is that there is no overlap with the CpGs identified based on SVA

^a^The FasT-LMM-EWASher method

^b^Unadjusted: cell type compositions were not included in the analyses

The motivation of adjusting for cell types was due to the potential confounding effects of cell type compositions with respect to the association of arsenic exposure with DNA methylation, caused by the association of arsenic exposure with cell type compositions [20–23]. Our assessment on the correlations between total arsenic exposure and estimated cell type proportions also supported the potential confounding-role of cell types (Additional file 1: Material S1). To support the existence of such confounding effects, we assessed the associations with and without adjusting for cell type proportions at all CpG sites. We found that at more than 99% of all the CpGs the effects (regression coefficients) of prenatal arsenic exposure changed by more than 10% when not adjusting for cell type (the median of the coefficients was 2.32 with 5^th^ percentile of 0.40 and 95^th^ percentile of 3.46) to adjusting for cell type (the corresponding statistics were 0.080, 0.0073 and 0.25), indicating a need of adjusting for cell types.

Overall, the analysis based on SVA identified CpG sites that had better overlap with the CpGs identified by other methods. To acquire the biological relevance of CpGs uniquely identified by use of SVA, we implemented DAVID to perform Gene Ontology (GO) analysis and to identify KEGG pathways. The 455 (out of 498) significant CpGs identified uniquely by SVA were mapped to genes using Illumina annotation file for 450 K DNA methylation array. Of great interest, GO categories related to transcription and regulation of RNA metabolic process were enriched after controlling FDR at 0.05, as well as three KEGG pathways, endocytosis, cancer pathway and MAPK signaling pathway (a complete list is included in Additional file 2: Material S2). A discussion on the connection of arsenic exposures and the identified GO categories and KEGG pathways is presented in the Discussion section.

### Findings from example data on cancer

We repeated the same analysis on an example data set provided by the FasT-LMM-EWASher package. A tutorial website for applying all the cell type composition inference methods to this example data is available at https://akhilesh362.wordpress.com/. This data set includes DNA methylation from the Illumina 27 K array and measures of a binary variable (cancer status) for 204 subjects. In total, 7648 CpGs were included in our study based on initial screening done by the FasT-LMM-EWASher package. The purpose of the initial screening is to exclude probes that are essentially not methylated or completely methylated. In this example data, cell type proportions were likely to be different on average between subjects with cancer and those without cancer, based on two-sample t-tests applied to logit-transformed sample proportions (Additional files 3 and 4: Materials S3 and S4), explaining the potential need to adjust for their confounding effects. Since Illumina 27 K focuses more on cancer genes, DNA methylation at a large number of CpG sites showed statistically significant associations with cancer status (Table 2). Some similar findings as in Table 1 were observed. ReFACTor identified a large number of CpGs, Fast-LMM-EWASher identified the least number of CpG sites, and SVA agreed nicely with minfi (Jaccard similarity index = 0.4). A unique observation from this analysis is that RUV identified the largest number of CpGs (6008 CpGs, close to the number of CpGs in the candidate pool, 7648 CpGs). Since the original Houseman et al. method was designed specifically for Illumina 27 K platform, it is understandable that SVA showed a better overlap with results from this approach (Jaccard similarity index = 0.4) compared to the results in the prenatal arsenic exposure and DNA methylation data. In total, 3 identified CpGs (cg22029275 located in the 1^st^ Exon of *FAM123A* gene, cg07080358 located in 1^st^ Exon of *CNRIP1*, and cg15202954 located within 200 base pair of transcription start site of *NALCN* gene) were common to all the eight cell correction methods as well as to the analyses without cell type composition adjusted. There is evidence that these three genes (*FAM123A, CNRIP1 and NALCN)* are associated with the risk of colorectal cancer [24–26].

**Table 2:** Number of significant CpG sites with and without cell-correction methods and overlap of CpG sites with those from the SVA method (example data from FasT-LMM-EWASher package)

Method	Identified CpGs (*N*)^#^	Overlap with SVA (%)	*p*-value^##^	J-index ^c^
Houseman et al.	1835	54.71	<0.0001	0.40
minfi	3589	84.59	<0.0001	0.40
SVA	1888	–	–	–
RefFreeEWAS	788	30.51	<0.0001	0.30
RefFreeCellMix	1006	18.38	<0.0001	0.10
ReFACTor	4224	87.45	<0.0001	0.40
EWASher ^a^	3	0.16	<0.0001	0
RUV	6008	99.95	<0.0001	0.30
Unadjusted ^b^	3768	82.89	<0.0001	0.40

^#^The selection of CpG sites is based on FDR-adjusted *p*-values (FDR is controlled at 0.05)

^##^ *P*-value is based on Fishers exact test for overlap. The null hypothesis is that there is no overlap with the CpGs identified based on SVA

^a^The FasT-LMM-EWASher method

^b^Unadjusted: cell type compositions were not incorporated into the analyses

^c^J-index is Jaccard index

DAVID analysis of genes associated with the significant CpGs identified uniquely by SVA led to the identification of three GO categories related to plasma membrane at FDR of 0.05 (integral to plasma membrane, intrinsic to plasma membrane, and plasma membrane part), as well as KEGG pathways such as pathways in cancer and signaling pathways (Additional file 2: Material S2), which indicates that genes corresponding to these CpG sites may play a role in the regulation of cancer.

### Findings from breast cancer status and DNA-methylation data

This analysis uses a data set discussed in Smith et al. [27]. Breast cancer status, DNA-methylation, and cell counts for granulocytes, monocyte, and lymphocytes for 61 subjects at baseline and a subset of 39 subjects at 6 months follow up are implemented in the analyses. Among all the methods discussed, the method implemented in the minfi package and the original Houseman et al. method were able to estimate cell proportions. We used minfi and the Houseman et al. approach to estimate the proportions of granulocyte, monocyte and lymphocyte cells. Lymphocyte proportions were derived by adding the proportions of B cell, T cell and Natural Killer (NK) cells. For the three cell types (granulocyte, monocyte and lymphocyte), Pearson correlations between estimated (minfi) and true cell proportions were 0.85, 0.79, 0.88 at baseline and 0.84, 0.78, 0.87 at the 6 month follow up, respectively. For the correlations based on the Houseman et al. method, they were 0.84, 0.78 and 0.88 at baseline and 0.78, 0.73 and 0.83 at the 6 month follow up, respectively. All the correlations showed statistically significant difference from zero (*p*-value < 0.05).

### Findings from simulated data

We simulated data applying two scenarios with the first scenario focusing on latent variable effects (comparable to effects of cell composition) and the second focusing on latent variable effects with confounding (comparable to effects of cell composition as well as confounding effects). In total, 100 data sets were simulated under each scenario. Details of the simulation scenarios are given in the Methods section. The simulated data were used to evaluate the five methods that do not estimate cell proportions nor need reference databases, specifically, FaST-LMM-EWASher, RefFreeEWAS, RefFreeCellMix, ReFACTor, and SVA.

For data under all scenarios, we applied each of the five methods to each simulated data to draw information on cell compositions. We then incorporated the information to assess the associations of “DNA methylation” with the variable of interest at each pseudo CpG site, and compared each method by assessing the sensitivity and specificity of the selected CpG sites across all 100 data sets. Regardless of the number of important CpGs, FaST-LMM-EWASher resulted in the lowest sensitivity but the highest specificity for both scenarios, consistent with findings from real data (Table 3). Findings from RefFreeEWAS, RefFreeCellMix, ReFACTor, and SVA are, in general, comparable for data simulated under scenario 1, but SVA gives consistently higher sensitivity and specificity in all settings (Table 3). For data simulated under scenario 2 with high correlations (*ρ* = 0.7), SVA outperformed FaST-LMM-EWASher, RefFreeEWAS, RefFreeCellMix and ReFACTor and had higher sensitivity and specificity. Compared with RefFreeEWAS, overall RefFreeCellMix outperformed when confounding effects were present, showing much higher sensitivities with relatively lower specificities. Results from ReFACTor indicated extremely low specificity under scenario 2, which is consistent with the rather large numbers of CpGs identified in real data. The performance of FaST-LMM-EWASher was similar between the two scenarios and was inferior to all other methods. On the other hand, the SVA method performed well under both scenarios, followed by RefFreeEWAS and RefFreeCellMix with RefFreeEWAS being weaker in capturing confounding effects. We also considered a situation with *ρ* = 0.3, mimicking a situation of moderate confounding (Additional file 5: Material S5), and similar patterns observed as those from the relatively two extreme cases (*ρ* = 0 and *ρ* = 0.7).

**Table 3:** Summary of sensitivity, specificity of Unadjusted, FaST-LMM-EWASher, RefFreeEWAS, SVA, ReFACTor and RefFreeCellMix for 100 simulated data across three settings

	Sensitivity (Median, 95% interval)		Specificity (Median, 95% interval)	
	Scenario 1 (*ρ* = 0)	Scenario 2 (*ρ* = 0.7)	Scenario 1 (*ρ* = 0)	Scenario 2 (*ρ* = 0.7)
	Number of Important CpGs =50			
Unadjusted	0.960 (0.470, 1.000)	1.000 (1.000, 1.000)	1.000 (0.987, 1.000)	0.000 (0.000, 0.000)
Ewasher ^a^	0.000 (0.000, 0.000)	0.000 (0.000, 0.000)	1.000 (0.999, 1.000)	1.000 (0.999, 1.000)
RefEWAS ^b^	1.000 (0.960, 1.000)	0.000 (0.000,0.494)	0.997 (0.994, 0.999)	0.579 (0.055,1.000)
CellMix ^c^	1.000 (0.980, 1.000)	1.000 (1.000, 1.000)	0.997 (0.993, 0.999)	0.546 (0.199, 0.923)
ReFACTor	1.000 (0.960, 1.000)	1.000 (1.000, 1.000)	0.996 (0.825, 1.000)	0.000 (0.000, 0.000)
SVA ^d^	1.000 (0.980, 1.000)	1.000 (0.960, 1.000)	0.998 (0.996, 1.000)	0.998 (0.996, 1.000)
	Number of Important CpGs =100			
Unadjusted	0.980 (0.664, 1.000)	1.000 (1.000, 1.000)	0.999 (0.976, 1.000)	0.000 (0.000, 0.000)
Ewasher ^a^	0.000 (0.000, 0.000)	0.000 (0.000, 0.000)	1.000 (0.999, 1.000)	1.000 (0.999, 1.000)
RefEWAS ^b^	1.000 (0.965,1.000)	0.000 (0.000,0.403)	0.995 (0.991, 0.998)	0.520 (0.014,1.000)
CellMix ^c^	1.000 (0.975, 1.000)	1.000 (1.000, 1.000)	0.988 (0.968, 0.996)	0.211 (0.047, 0.525)
ReFACTor	1.000 (0.965, 1.000)	1.000 (1.000, 1.000)	0.994 (0.808, 0.998)	0.000 (0.000, 0.000)
SVA ^d^	1.000 (0.990,1.000)	0.990 (0.965, 1.000)	0.996 (0.993, 0.999)	0.996 (0.993, 0.999)
	Number of Important CpGs =150			
Unadjusted	0.993 (0.723, 1.000)	1.000 (1.000, 1.000)	0.999 (0.965, 1.000)	0.000 (0.000, 0.000)
Ewasher ^a^	0.000 (0.000,0.000)	0.000 (0.000,0.000)	1.000 (0.999,1.000)	1.000 (0.999,1.000)
RefEWAS ^b^	0.993 (0.973,1.000)	0.000 (0.000,0.294)	0.992 (0.986, 0.997)	0.496 (0.013,1.000)
CellMix ^c^	1.000 (0.983, 1.000)	1.000 (1.000, 1.000)	0.975 (0.929, 0.993)	0.098 (0.022, 0.293)
ReFACTor	1.000 (0.980, 1.000)	1.000 (1.000, 1.000)	0.989 (0.794, 0.997)	0.000 (0.000, 0.000)
SVA ^d^	1.000 (0.993,1.000)	0.993 (0.970, 1.000)	0.992 (0.988, 0.996)	0.993 (0.988, 0.996)

*ρ* = correlation between primary covariate and latent variables

*ρ* = 0 corresponds to data simulated from Scenario 1, while *ρ* = 0.7 corresponds to data simulated from Scenario 2

^a^FasT-LMM-EWASher

^b^RefFreeEWAS

^c^RefFreeCellMix

^d^Surrogate variable analysis

**Fig. 2:**
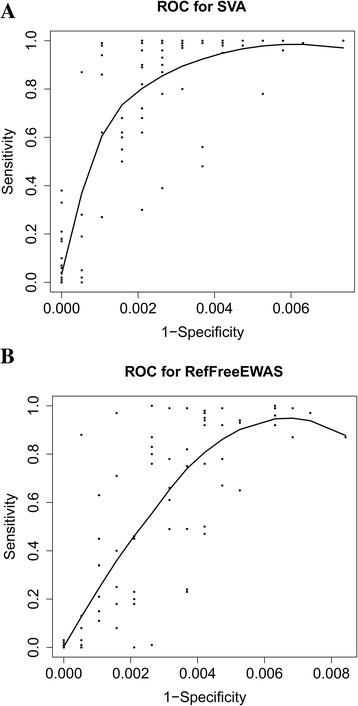
Plots of sensitivity vs. 1-specificity and estimated ROC curves, **a**) SVA. **b**) RefFreeEWAS

In the above simulations, we fixed the regression coefficients of the important CpGs. To demonstrate the pattern of sensitivity and specificity, we implemented receiver operating characteristic (ROC) plots. In total, 100 data sets were simulated under scenario 1 with regression coefficients for the variable of interest ranged from 0.01 to 0.3. For each data set, we calculated sensitivity and specificity of selected CpGs, based on which we estimated the ROC curves. Sensitivities from FaST-LMM-EWASher were substantially low and were not considered in this demonstration. The performance of RefFreeEWAS, RefFreeCellMix, and ReFACTor was comparable under scenario 1 (Table 3). We therefore only presented ROC curves for ReFreeEWAS and SVA for the purpose of comparison (Fig. 2). The findings are consistent with what we observed from Table 2 for scenario 1, that is, SVA performed better than RefFreeEWAS. In addition, the results indicated that both SVA and RefFreeEWAS have high specificity regardless of the underlying regression coefficients, indicating the conservativism when selecting informative CpGs.

## Discussion

We compared eight cell-type correction methods using real and simulated data. Based on DNA methylation in a cohort study, the methods in ReFACTor identified the largest number of CpGs (~60 K CpGs), none of which overlapped with the common CpGs detected by other methods including the analysis without adjusting for cell type compositions (but excluding the method in FaST-LMM-EWASher). The method in FaST-LMM-EWASher did not identify any CpG sites. Except for ReFACTor and FaST-LMM-EWASher, at least one detected CpG was shared between all the other methods. More than 50% of CpGs identified by the Houseman et al. method and by the approach implemented in minfi were also detected by the SVA method; The overlap in CpGs was much less between these two methods and the remaining methods. The genes associated with CpGs uniquely identified by using SVA with prenatal urinary arsenic as primary exposure led to the enrichment of GO categories and KEGG pathways that were consistent with our understanding with respect to the effect of arsenic on DNA methylation. Arsenic exposure leads to generation of reactive oxygen species (ROS) which induces DNA damage [28]. This reactive oxygen species play a crucial role in signal transduction pathways, transcription factor regulation [29], and mitogen activated protein kinases (MAPKs) signal transduction pathway is one such pathway that is affected by ROS [30]. DAVID analysis of genes associated with the CpGs uniquely identified by SVA for FaST-LMM-EWASher example dataset led to enrichment of KEGG pathways in cancer. All these imply that the CpGs uniquely identified by using SVA are potentially informative. Using the example dataset provided by FasT-LMM-EWASher method, we found that all methods except for FasT-LMM-EWASher identified a large number of CpG sites. This was likely due to the platform used to measure DNA methylation levels (Illumina 27 K), which is centered more on cancer genes. However, CpGs identified based on ReFACTor and RUV were close to the number of CpGs in the pool of candidate CpGs, indicating possible inflations. On the other hand, results from minfi showed the greatest overlap with the SVA method (Table 2). Based on these two real data sets, results from the method in the minfi package and those from SVA were most agreeable. However, for real data, the underlying truth was unknown, which was the motivation of incorporating a data set with cell counts known and the use of a series of simulation studies. Findings from these data were further discussed in this section.

Using the available cell counts in the cancer status and DNA methylation dataset we observed agreements between cell types estimated by Houseman et al. and minfi, but minfi showed a better agreement. The Houseman et al. approach was designed for the Illumina 27 K beadchip array, which may not fit the 450 K array as noted in the literature [4]. The modification of the Houseman et al. approach implemented in the minfi package, on the other hand, is suitable for both 27 K and 450 K array. The reference data were from six adult white European males. It has been shown that DNA methylation patterns vary by sex, age and ancestry [31–35]. Generalizing the cell mixtures estimated by minfi to studies with both genders and non-Europeans of different age groups may potentially introduce bias.

Further simulations investigating reference-free methods supported the findings from real data. Regardless of the number of important CpGs, FaST-LMM-EWASher showed the lowest sensitivity, indicating low power to identify truly important CpGs if using that method to adjust for cell type compositions. ReFACTor produced the lowest specificity when confounding effects were present, supporting the rather low overlapping with findings from other methods. On the other hand, findings from ReFACTor, RefFreeEWAS, RefFreeCellMix and SVA were in general comparable for data simulated under scenario 1 (no-confounding effects) but SVA gave consistently higher sensitivity and specificity when cofounding effects present.

The SVA approach does not provide estimates on cell type compositions; however, our ultimate goal was not to estimate cell counts. The goal was to identify an approach that best assesses DNA methylation differentiation due to exposure or diseases, corrected for a potential cell type bias. From this viewpoint and the findings from real data and the high sensitivities and specificities from simulations (under both scenarios, confounding and no confounding), using SVA to adjust for cell type compositions seems to be an appropriate method and may perform better than other existing methods. After including two additional methods (ReFACTor and RefFreeCellMix) and implementing more stringent conditions of confounding in simulations (by assuming higher correlations and dynamic correlations in nearby CpGs), we reached the same conclusion as in McGregor et al. [12] when the focus was on assessing associations. We would like to point out that since the method in minfi focuses on estimating cell type compositions, it does not have the ability to address variations in any unknown factors. Thus, it is expected that this approach will not outperform the SVA approach in association studies, as found in McGregor et al., although the method in minfi provides better estimates on cell type proportions. It is also worth noting that information included in the surrogate variables produced by the SVA method may also include other information in addition to cell type compositions. There is a potential of over-adjustment by use of this approach. Furthermore, we note that all these reference-free methods can be directly applied to genome-wide bisulfite sequencing data and we expect similar findings in terms of their ability in inferring cell type compositions.

## Conclusions

When appropriate reference data are available and if inferences on cell type compositions are needed, the method implemented in the minfi package is recommended. However, if no such reference data are available or if the focus is not on estimating cell proportions, the SVA method is suggested to correct for bias resulting from varying cell mixtures, the same conclusion given by McGregor et al. [12].

## Methods

In this section we briefly describe the existing techniques for estimating cell proportions or inferring latent variables due to cell compositions, data sets (real and simulated) to assess these methods, and statistical methods used in the analyses. All the analyses were programmed in R and a tutorial website including all the programs demonstrating the methods is available at https://akhilesh362.wordpress.com/, and can also be accessed via http://www.memphis.edu/sph/people/faculty_profiles/zhang.php.

### Existing methods for cell compositions

- *Reference-based methods*: Houseman et al. [3] developed a method for cell type correction that capitalizes on the idea that differentially methylated regions (DMRs) can serve as a signature for the distribution of different types of white blood cells. It uses these DMRs as a surrogate in a regression calibration based technique to identify the cell mixture distribution. Regression calibrations can lead to bias estimates, thus an external validation data is used to calibrate the model and to correct for the bias [36]. Their method was specifically for the Illumina 27 k beadchip array (Illumina, Inc., San Diego, CA, USA).
  The method by Jaffe and Irizarry [4] was adapted from the Houseman et al. [3] method and is tailored for Illumina450k along with 27 k array. The algorithm in Houseman et al. identified 500 CpG sites used to estimate cell mixture proportions from the Illumina 27 k array. The modification of Jaffe and Irizarry was motivated because of the existence of probe SNPs in the 500 CpG sites and the inconsistency of CpG sites between the 27 k and 450 k arrays. In addition, the flow-sorted data of the six adult male subjects were used as references [37] when DNA methylation was measured in peripheral blood. For DNA methylation in cord blood, cord blood reference data were used [38].
  The method of removing unwanted variation (RUV) uses information from a reference database, but it does not estimate cell type proportions. Instead, this approach is based on the information on negative control probes and performs factor analysis on these probes to identify factors due to unmeasured confounders. These factors are then included in subsequent analyses to adjust for cell type effects. The negative control probes were chosen as the top 500 CpG sites from the reference database of DNA methylation known to be correlated with the cell types [39].
- *Reference-free methods*: In total, four commonly used or recently developed reference-free methods are implemented in our study, FaST-LMM-EWASher, RefFreeEWAS, RefFreeCellMix, and ReFACTor. These methods do not need any external validation datasets and have the potential to adjust for cell mixture arising from any tissue, including blood. FaST-LMM-EWASher [40] applies the maximum likelihood (ML) approach in linear mixed models and optimizes spectral decomposition to estimate cell types [41]. RefFreeEWAS utilizes singular value decomposition (SVD) to decompose the residuals of unadjusted linear models along with unadjusted linear coefficient estimates, and estimates latent subject and cell-specific effects. Bootstrap estimates for coefficient standard errors are used to account for the correlation in the error structure.
  Surrogate variable analysis (SVA) estimates potential confounding factors from a singular value decomposition (SVD) of residuals and was initially applied to gene expression data [42]. SVA utilizes the concept of expression heterogeneity while estimating surrogate variables. Expression heterogeneity (EH) refers to certain plausible biological profiles of the subject, which may not be captured by the covariates in study. Compared to the method in RefFreeEWAS, SVA decomposes the residual matrix and utilizes permutations to identify statistically significant eigen-vectors which serve as a representative of EH (the so-called eigengenes), and then infers surrogate variables based on theses “eigengenes”. Surrogate variables from SVA have the potential to cover information on cell types in DNA methylation from blood cells.
  The method built in the R package RefFreeCellMix is improved from that in RefFreeEWAS. It uses a variant of non-negative matrix factorization to decompose the total methylation sites into CpG-specific methylation states for a pre-specified number of cell types and subject-specific cell-type distributions [8]. Another approach in the R package, ReFACTor, implements a variant form of principal component analysis (PCA) to adjust for the cell type effects. This method assumes that a small number of methylation sites are affected by underlying cell mixtures. It filters out CpGs if the variation is not large enough (the default cutoff is standard deviation = 0.02). To avoid filtering out too many CpGs, in our analyses, we excluded CpGs such that their standard deviations were in the lower 5^th^ percentile. By default this method searches for top 500 most informative methylation sites and performs PCA with a fixed number of components on these CpG sites to obtain the components. These ReFACTor components can be used as a covariate in epigenome wide association study or can be added one at time to remove the inflation due to cell type composition [10].

### Three real data sets used to compare the approaches

These three data sets include data on prenatal arsenic exposure and DNA methylation, an example data from FaST-LMM-EWASher, and data on breast cancer status and DNA methylation. The first two data sets were utilized to demonstrate each of the five methods for cell type compositions and their agreement in terms of identified CpGs potentially associated with a variable of interest. The third data set was used to assess the agreement between the estimated cell type proportions (using the Houseman et al. method and the method in minfi) and the physical counts of the cells. This data set served as a benchmark and was critical for the comparison between the Houseman et al. method and the method in minfi. The benchmark data used to compare reference-free methods were simulated data, as discussed in the next section.

- *Prenatal arsenic exposure and DNA methylation data*: The data were from a birth cohort study examining multiple prenatal and postnatal factors in relation to child health outcomes, part of the nationwide Taiwan prenatal and infant cohort study [13, 14] established in Taiwan in 2000–2001. In total, 64 subjects with genome-scale DNA methylation measured in cord blood and levels of prenatal arsenic exposure were included in our study. DNA methylation data were pre-processed including quantile normalization, probe-type correction, and probe SNPs exclusion. After pre-processing, in total, 385,183 CpG sites were included in the analyses. All the five methods were applied to this data set. This and the following example data set were used to compare the performance of the five methods.
- *An example cancer data from FaST-LMM-EWASher*: This is an example data provided by the FaST-LMM-EWASher package [43]. It was originally used to illustrate the method incorporated in FaST-LMM-EWASher. In total, 204 subjects with cancer status and DNA methylation from Illumina 27 K array on 25,978 CpG sites are available.
- *Breast cancer status and DNA methylation data*: This data set has been previously described [27] and has genome-scale DNA methylation and breast cancer status available on 61 subjects at baseline and on 39 subjects at 6 month follow-up along with complete blood counts. After pre-processing, 484,489 CpG sites were included in the study. In this article, we focus on granulocytes, monocyte and lymphocytes cells since proportions of these cells can be estimated by use of the minfi package and the original Houseman et al. approach. In our study, proportions of these cells from the physical counts were compared to the cell proportions estimated by minfi and the Houseman et al. method.
  Both studies (the arsenic and DNA methylation related study and the breast cancer and DNA methylation related study) were approved by internal review board. Nurses and doctors were involved in the data collections. None of the authors were involved in data collection and handling. The data used in this analysis were de-identified.

### Simulated data sets to compare the approaches

To further evaluate the three reference-free methods (FaST-LMM-EWASher, RefFreeEWAS, RefFreeCellMix, ReFACTor, and SVA), we simulated DNA methylation data under different settings with “latent” variables representing “cell types”. These data sets served as benchmark data for comparing reference-methods because the underlying truth was known. Two simulation scenarios were employed to evaluate the methods.

- *Scenario 1*: We simulated DNA methylation data at 2000 CpG sites across 600 samples, of which the first *n* CpG sites were associated with covariates of interest (e.g., level of arsenic exposure) and a set of latent variables, and the remaining CpG sites were only associated with the latent variables. The set of latent variables represent “cell types”. One covariate of interest was considered and generated from a Normal distribution with mean 0 and variance 1 (*N*(0, 1)), The coefficient of this covariate was set at 0.3 and the intercept in the regressions was set to 0.5. Five “latent” variables were used and generated from five different Normal distributions: *N*(0,5), *N*(3,1), *N*(0,1), *N*(2,4), *N*(0,3), respectively. The association of DNA methylation and the latent variables was assumed linear and the coefficients were generated from *N* (0.5, 0.01). The distribution of random errors in the linear regressions was assumed to be Normal with mean 0 and variance 1.2 for the *n* CpGs, mean 0 and variance of 1.2 for the next 100 CpGs, and mean 0 and variance 2 for the remaining CpGs. The last setting with larger variance in random errors was for situations where the influence of cell types on DNA methylation was weaker.
  We took three values of *n*, *n* = 50, 100, and 150, representing different sparsity levels (from high to low) of informative CpGs. In total, 100 data sets for each *n* were simulated. Note that under this scenario, the covariates and latent variables were generated separately and had no correlations.
- *Scenario 2*: Latent variables generated under this scenario have potential confounding effects. The overall setting is the same as in scenario 1, except that the covariate of interest and the five latent variables (6 variables in total) were correlated such that correlation is equal to 0.7^*|i-j|*^, *i, j* = 1, 2, 3, 4, 5, 6. For instance, the correlation of the continuous covariate with the first latent variable was 0.7, and with the second latent variable was 0.7^2^ = 0.49.

### The flow of the analyses plan

The overall flow of the analyses plan is as follows: Step 1. We applied all the eight methods to two real data sets (the prenatal arsenic exposure and DNA methylation data and the example cancer data from FaST-LMM-EWASher) to assess the agreement within each data set in terms of identified CpGs between different methods, assess the consistency of each method between different data sets, and use existing knowledge and tools to qualitatively compare each approach. Step 2. We used the breast cancer status and DNA methylation data which had cell counts available to quantitatively compare the two reference-based approaches (the method in minfi and the method by Houseman et al.) in terms of their agreement with the true cell counts. Step 3. We implemented simulated data to compare the reference-free methods based on sensitivities and specificities. The inability of quantitative comparison in Step 1 (where the underlying truth was unknown) motivated the subsequent comparisons in Steps 2 and 3 (underlying truth was known).

### Statistical analyses

Linear regression-based analyses were used to assess the associations of DNA methylation with variables of interest with cell type heterogeneity adjusted using eight different methods. In the analyses of the two real data sets (the arsenic and DNA methylation data, and the FasT-LMM-EWASher example data), we recorded CpG sites showing statistically significant association with variables of interest (i.e., arsenic exposure and cancer) after implementing different cell type heterogeneity inference methods. We also inferred the number of statistically significant CpG sites without adjusting for cell type heterogeneity. To compare the eight cell type heterogeneity inference methods (Houseman et al., minfi, FaST-LMM-EWASher, RefFreeEWAS, RefFreeCellMix, ReFACTor, RUV, and SVA), we assessed the percentage of overlap between different methods in the number of identified CpG sites that showed statistical significance, and calculated a similarity index, Jaccard index (J-index) [44]. The percentage of overlap is calculated as the number of identified CpGs overlapped with that from SVA divided by the number of CpGs identified by SVA. We used Fisher exact test to assess the significance of overlap. Jaccard index measures the similarity between two finite sample sets. We used a Bioconductor package GeneOverlap to calculate this index. To assess whether the CpGs uniquely identified by the SVA approach were informative, we used the Database for Annotation, Visualization and Integrated Discovery (DAVID) [45, 46] to analyze the enrichment in Gene ontology (GO) [47] categories and Kyoto Encyclopedia of Genes and Genomes (KEGG) [48, 49] pathways.

As for each simulated data set, we calculated sensitivity and specificity of the selected CpG sites for each cell type heterogeneity inference method. They were calculated by comparing the detected CpGs with the truly important CpGs. For each of the five methods, median of sensitivity and specificity along with 95% empirical intervals across 100 data sets were recorded for each setting under each simulation scenario.

## Additional files

Additional file 1: Supplemental Material S1. Figure A. Pearson correlations between inorganic arsenic levels (in log10 scale) and cell type proportions. Figure B. Pearson correlations between total arsenic levels (in log10 scale) and cell type proportions. (PDF 268 kb)

Additional file 2: Supplemental Material S2. Functional annotation of genes related to CpGs identified by SVA method for the Taiwanese and example data in the FasT-LMM-EWASher package. (PDF 9 kb)

Additional file 3: Supplemental Material S3. *T*-test results for differences in cell proportions for six cell types across cases and control (cancer status). (PDF 7 kb)

Additional file 4: Supplemental Material S4. Boxplots depicting the difference in proportions for six cell types across cases and control (cancer status). (PDF 795 kb)

Additional file 5: Supplemental Material S5. Summary of sensitivity, specificity of Unadjusted, FaST-LMM-EWASher, RefFreeEWAS, SVA, ReFACTor and RefFreeCellMix for 100 simulated data across three settings for *ρ* = 0, 0.3 and 0.7. (PDF 418 kb)

## Abbreviations

CpG: Cytosine phosphate guanine
DAVID: Database for Annotation, Visualization and Integrated Discovery
DMRs: Differentially methylated regions
FDR: False discovery rate
GO: Gene ontology
KEGG: Kyoto Encyclopedia of Genes and Genomes
ROS: Reactive oxygen species
SNPs: Single nucleotide polymorphism
SVA: Surrogate variable analysis
