# Supplementary material for: Comparison of different cell type correction methods for genome-scale epigenetics studies
Source: BMC Bioinformatics. 2017 Apr 14;18:216. doi: 10.1186/s12859-017-1611-2 (PMC5391562; doi:10.1186/s12859-017-1611-2)
Supplement: Supplementary file 1 — Supplemental Material S1. Figure A. Pearson correlations between inorganic arsenic levels (in log10 scale) and cell type proportions. Figure B. Pearson correlations between total arsenic levels (in log10 scale) and cell type proportions. (PDF 268 kb) [file 12859_2017_1611_MOESM1_ESM.pdf]

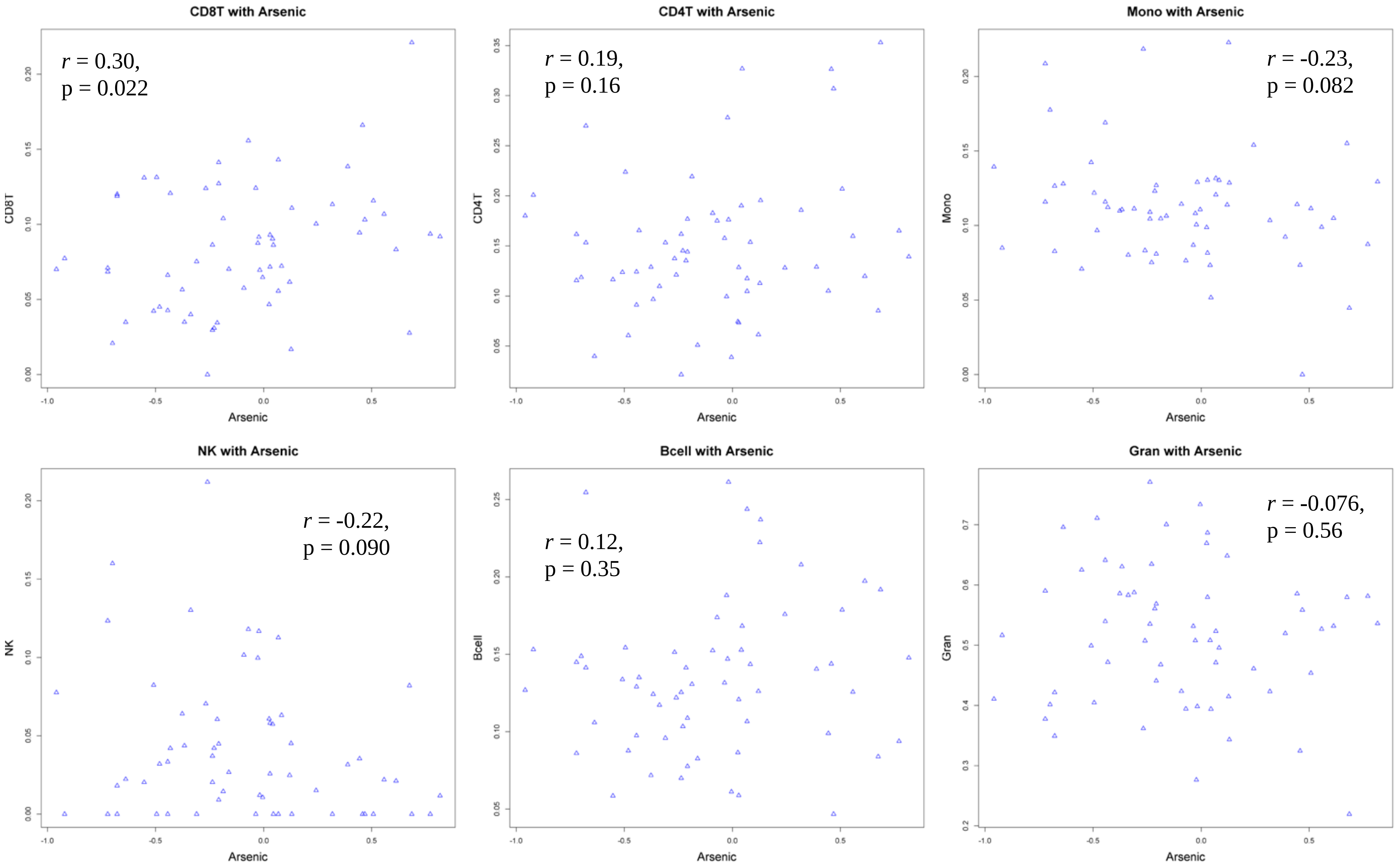

Figure A. Pearson correlations between **inorganic arsenic levels** (in log10 scale) and cell type proportions

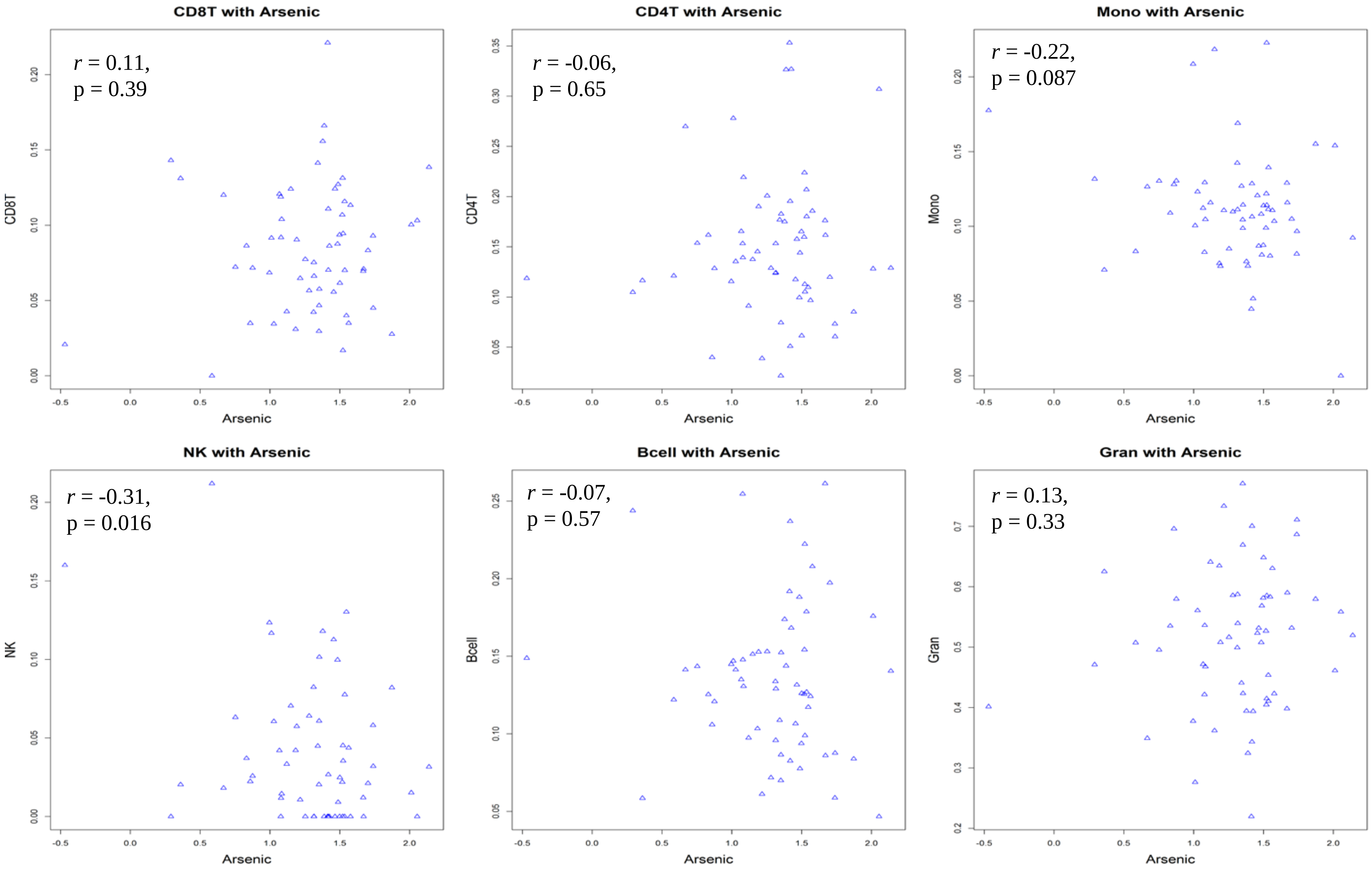

Figure B. Pearson correlations between **total arsenic levels** (in log10 scale) and cell type proportions.
