# Supplementary material for: Comparison of different cell type correction methods for genome-scale epigenetics studies
Source: BMC Bioinformatics. 2017 Apr 14;18:216. doi: 10.1186/s12859-017-1611-2 (PMC5391562; doi:10.1186/s12859-017-1611-2)
Supplement: Supplementary file 2 — Supplemental Material S2. Functional annotation of genes related to CpGs identified by SVA method for the Taiwanese and example data in the FasT-LMM-EWASher package. (PDF 9 kb) [file 12859_2017_1611_MOESM2_ESM.pdf]

Supplemental Material 2

A complete list of enriched GO categories and KEGG pathways statistically significant at false discovery rate of 0.05.

1) **Prenatal arsenic exposure and DNA-methylation data**
   GO categories: (1) transcription, (2) regulation of transcription, (3) regulation of RNA metabolic process, and (4) DNA binding
   KEGG pathways: endocytosis, cancer pathway and MAPK signaling pathway.
2) **An example data by the FasT-LMM-EWASher package**
   GO categories: (1) integral to plasma membrane, (2) intrinsic to plasma membrane, (3) plasma membrane part.
   KEGG pathways: (1) Pathways in cancer, (2) MAPK signaling pathway, (3) Regulation of actin cytoskeleton, (4) Neuroactive ligand-receptor interaction, (5) Cell adhesion molecules (CAMs), (6) Calcium signaling pathway, (7) Cytokine-cytokine receptor interaction, (8) Jak-STAT signaling pathway, (9) Hematopoietic cell lineage, (10) Complement and coagulation cascades, and (11) Melanoma.
