# Supplementary material for: Comparison of different cell type correction methods for genome-scale epigenetics studies
Source: BMC Bioinformatics. 2017 Apr 14;18:216. doi: 10.1186/s12859-017-1611-2 (PMC5391562; doi:10.1186/s12859-017-1611-2)
Supplement: Supplementary file 3 — Supplemental Material S3. T-test results for differences in cell proportions for six cell types across cases and control (cancer status). (PDF 7 kb) [file 12859_2017_1611_MOESM3_ESM.pdf]

The test results comparing cell type proportions between cases and controls (based on two sample t-tests applied to logit transformed cell type proportions). This is for the example data from the FasT-LMM-EWASher package (Diff.: difference in means of logit transformed cell type proportions between controls and cases). Boxplots of cell type proportions of cases and controls for each cell type are on the next page.

| | **CD8T** | **CD4T** | **NK** | **Bcell** | **Mono** | **Gran** |
|---|---|---|---|---|---|---|
| **Diff.** | 7.811346 | 0.1108 | -15.7326 | -6.66479 | 8.899697 | 4.970291 |
| **P-value** | $2.10\times10^{-11}$ | 0.911954 | $1.70\times10^{-35}$ | $3.50\times10^{-10}$ | $3.88\times10^{-12}$ | $1.96\times10^{-6}$ |
