# Supplementary figures and images for: Comparison of different cell type correction methods for genome-scale epigenetics studies
Source: BMC Bioinformatics. 2017 Apr 14;18:216. doi: 10.1186/s12859-017-1611-2 (PMC5391562; doi:10.1186/s12859-017-1611-2)

CD8T

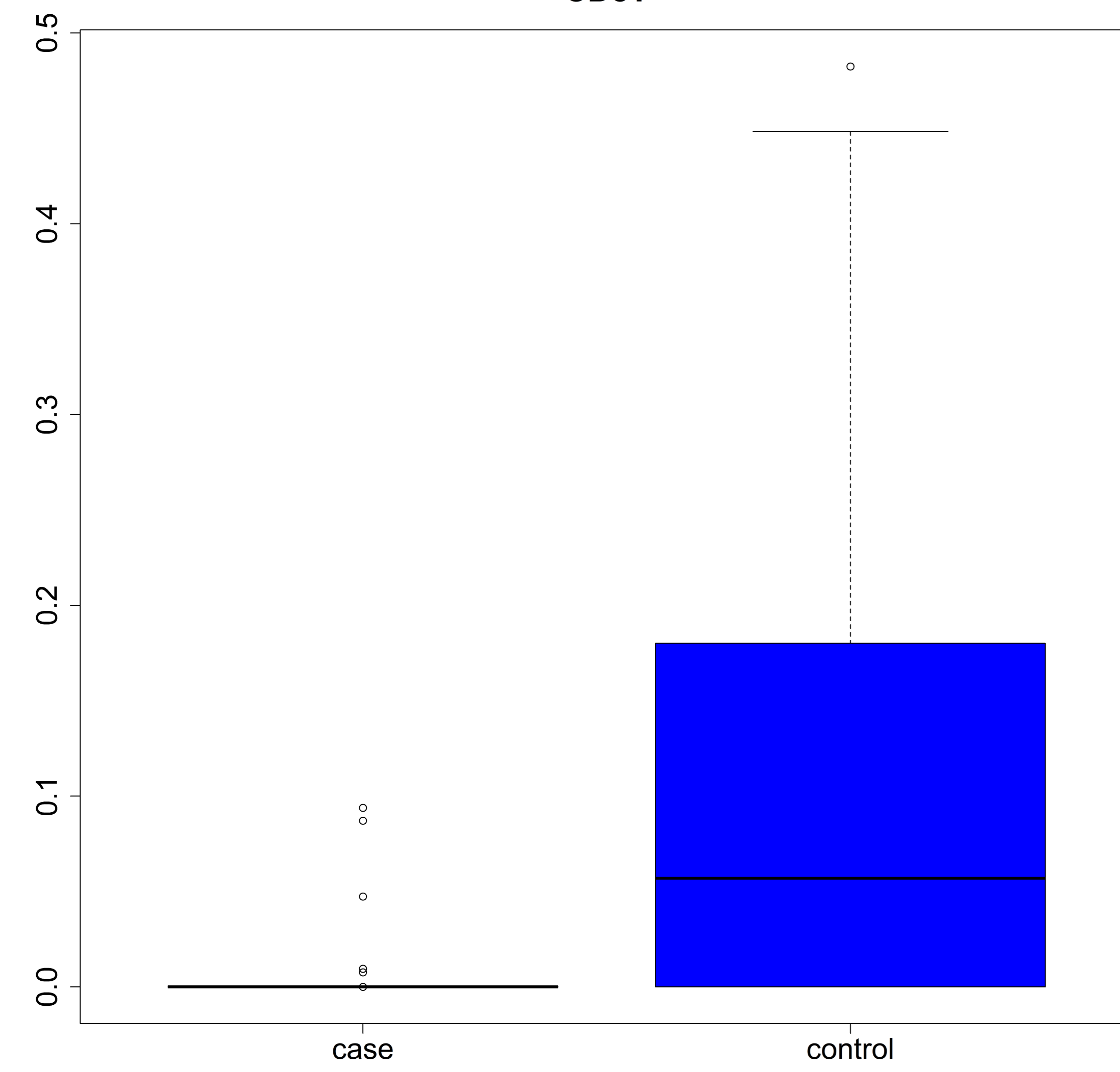

CD4T

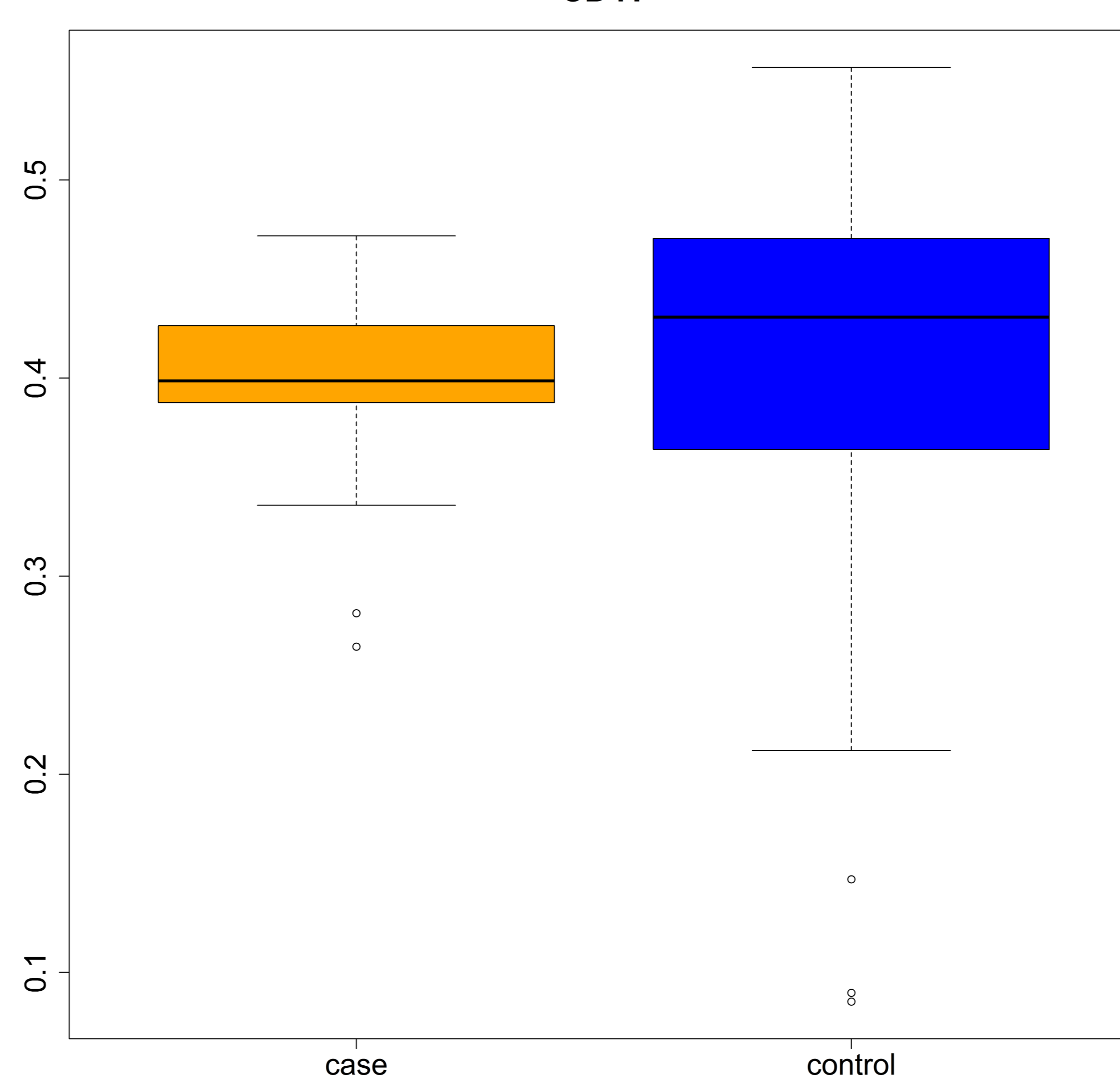

Mono

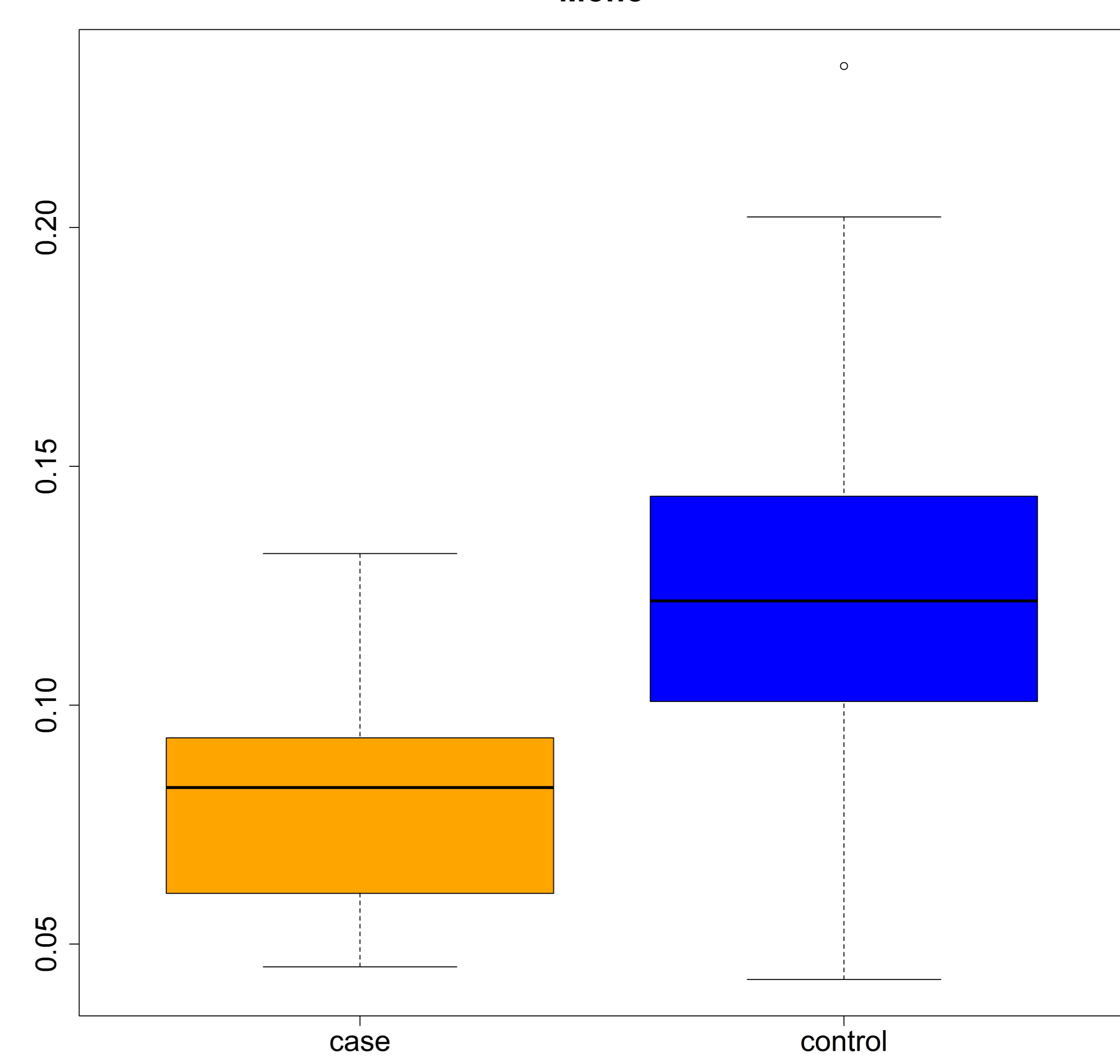

NK

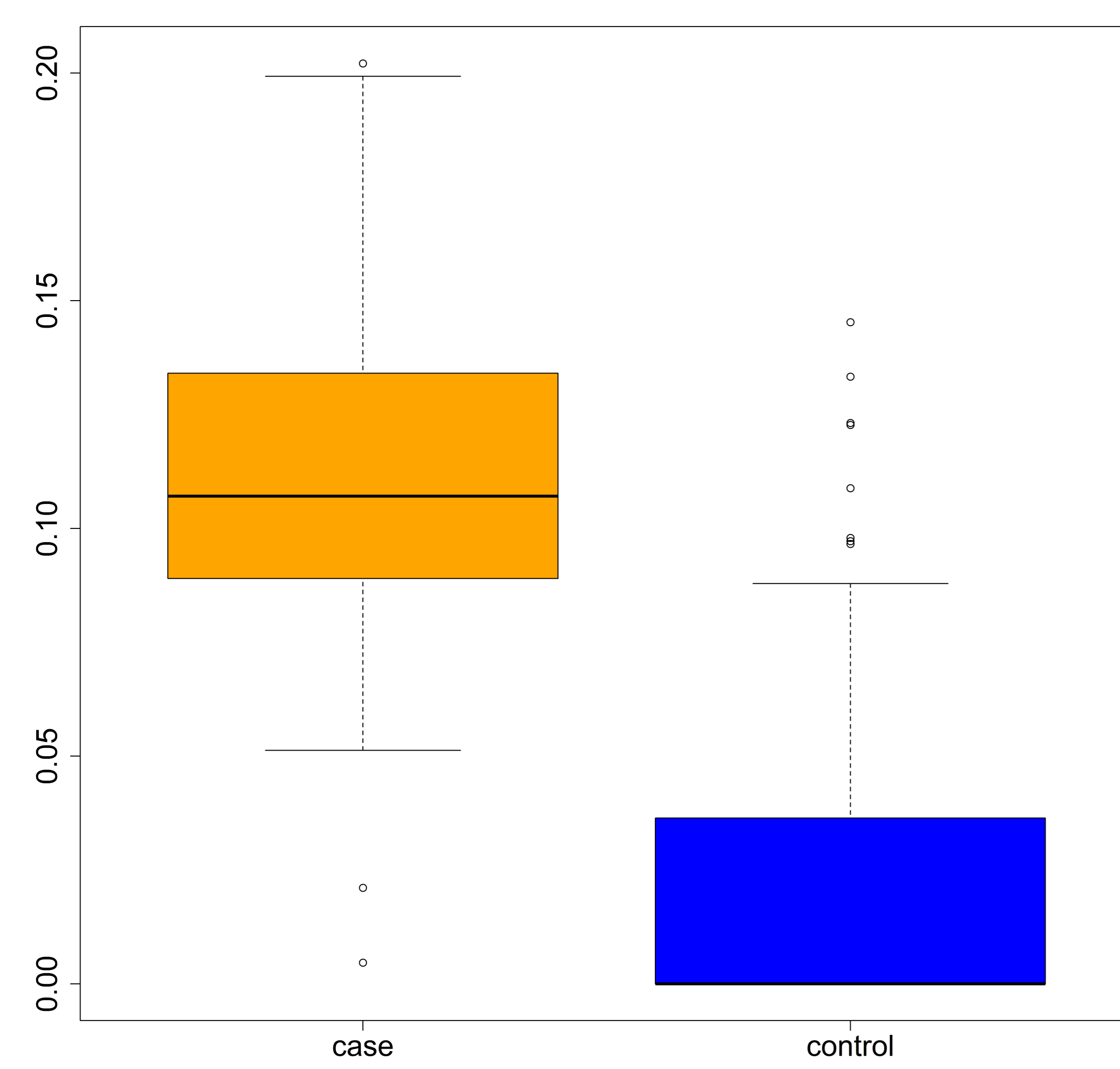

Gran

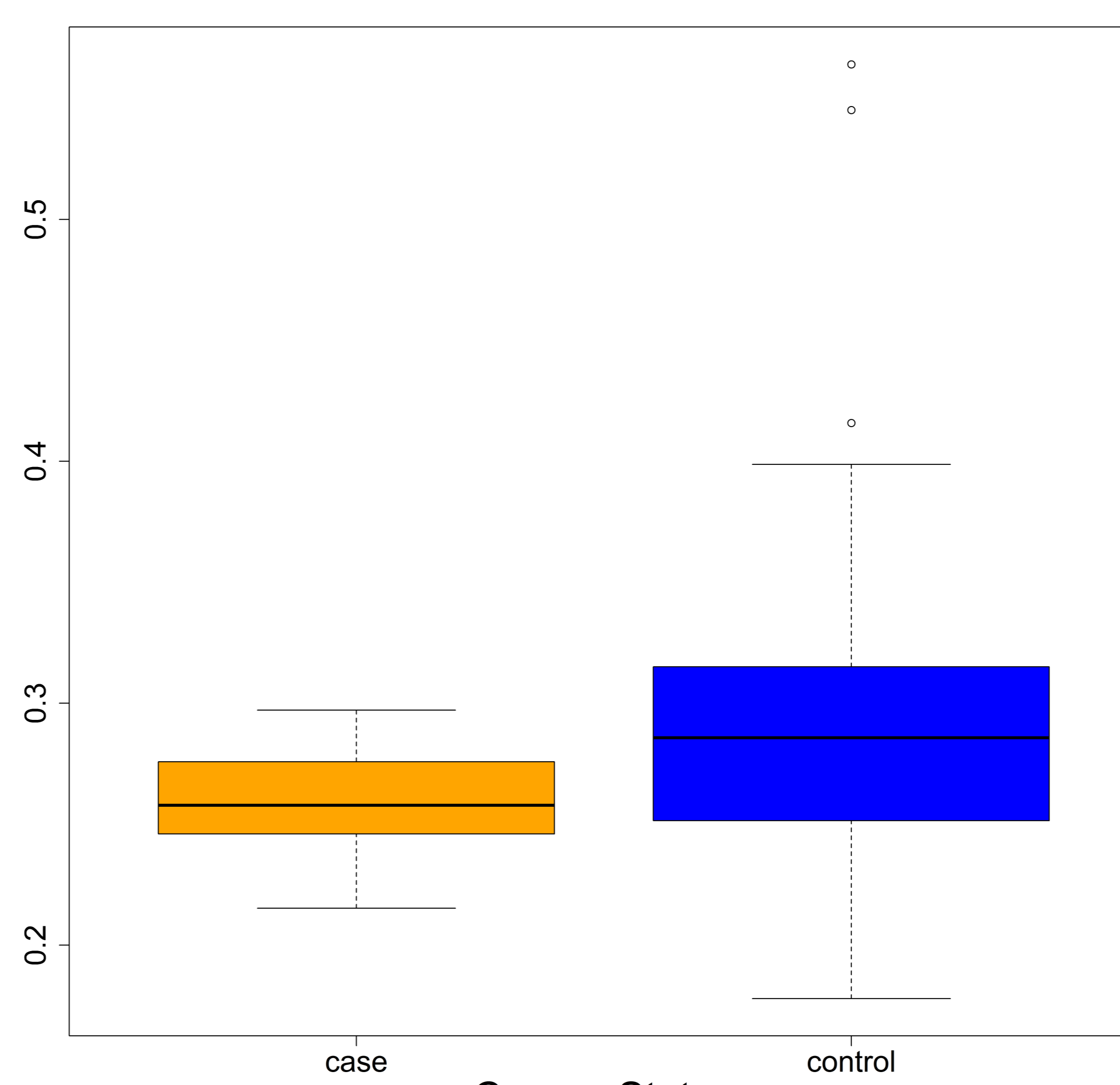

Bcell

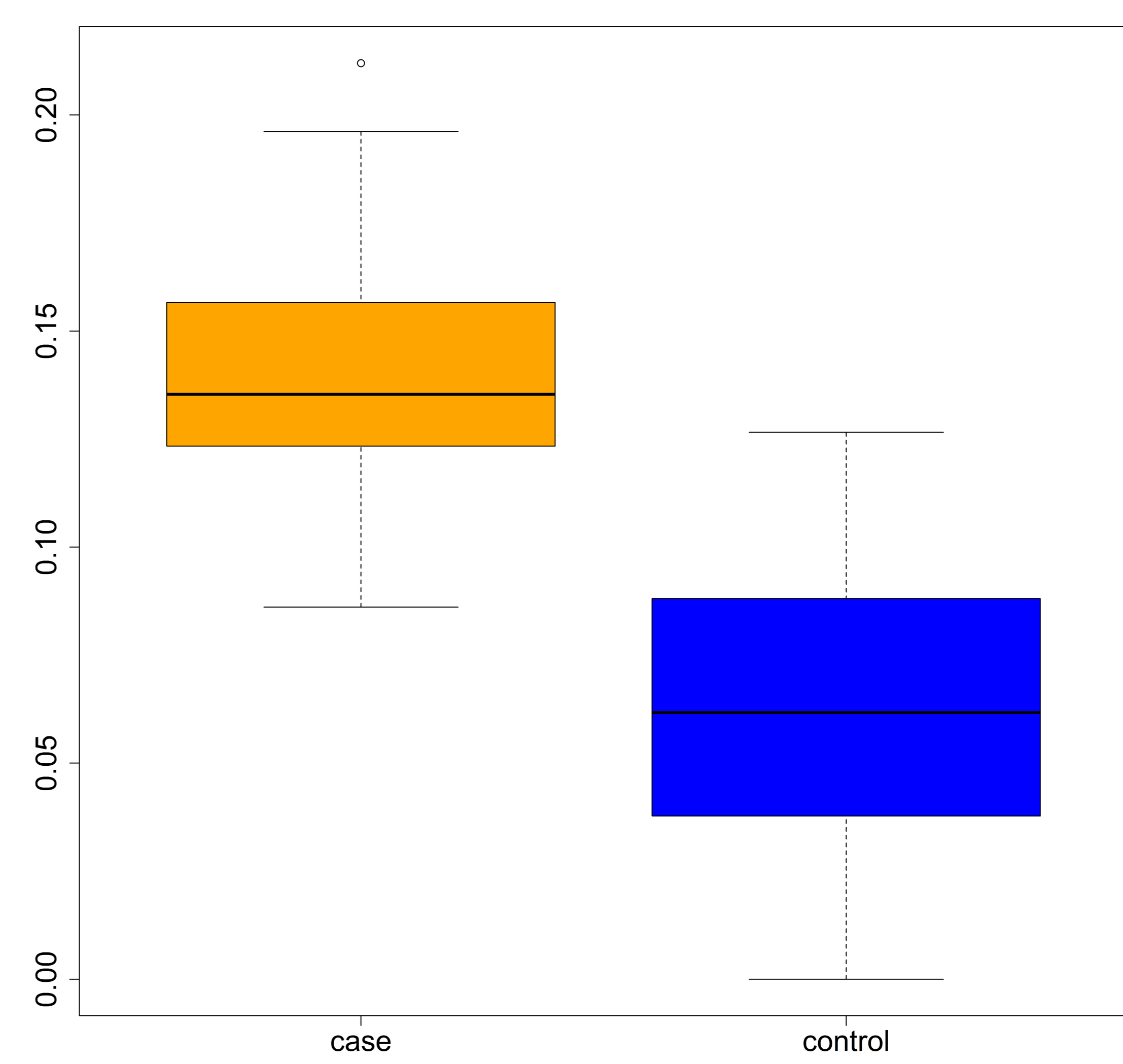

Cancer Status

Supplement: Supplementary file 4 — Supplemental Material S4. Boxplots depicting the difference in proportions for six cell types across cases and control (cancer status). (PDF 795 kb) [file 12859_2017_1611_MOESM4_ESM.pdf]
