# Supplementary material for: Comparison of different cell type correction methods for genome-scale epigenetics studies
Source: BMC Bioinformatics. 2017 Apr 14;18:216. doi: 10.1186/s12859-017-1611-2 (PMC5391562; doi:10.1186/s12859-017-1611-2)
Supplement: Supplementary file 5 — Supplemental Material S5. Summary of sensitivity, specificity of Unadjusted, FaST-LMM-EWASher, RefFreeEWAS, SVA, ReFACTor and RefFreeCellMix for 100 simulated data across three settings for ρ = 0, 0.3 and 0.7. (PDF 418 kb) [file 12859_2017_1611_MOESM5_ESM.pdf]

Supplemental Material S5.

Summary of sensitivity, specificity of FaST-LMM-EWASher, RefFreeEWAS, RefFreeCellMix, ReFACTor, SVA, as well as results when "latent factors" were not adjusted. Results were based on 100 simulated data across three settings (ρ=0, 0.3, and 0.7) each with different numbers of important CpGs (50, 100, and 150).

	Number of Important CpGs =50						
	**Sensitivity**(Median, 95% interval)				**Specificity**(Median, 95% interval)		
	**ρ=0**	**ρ=0.3**	**ρ=0.7**		**ρ=0**	**ρ=0.3**	**ρ=0.7**
**Unadjusted**	0.96(0.47, 1)	1(1,1)	1(1, 1)		1(0.987, 1)	0.21(0.00, 0.99)	0(0, 0)
**Ewasher**	0(0, 0)	0(0, 0)	0(0, 0)		1(0.999, 1)	1(0.999, 1)	1(0.999, 1)
**RefFreeEWAS**	1(0.96, 1)	0.982(0.437, 1)	0(0, 0.494)		0.997(0.994, 0.999)	0.996(0.993, 0.999)	0.579(0.055, 1)
**SVA**	1(0.98, 1)	1 (0.98, 1)	1(0.96, 1)		0.998(0.996, 1)	0.998 (0.996, 1)	0.998(0.996, 1)
**Refactor**	1(0.96, 1)	1(1,1)	1(1, 1)		0.996(0.825, 1)	0.042 (0, 0.711)	0(0, 0)
**RefFreeCellMix**	1(0.98, 1)	1(1, 1)	1(1, 1)		0.997(0.993, 0.999)	0.983(0.930, 0.997)	0.546(0.199, 0.923)
	**Number of Important CpGs =100**						
**Unadjusted**	0.98(0.664, 1)	1(1, 1)	1(1, 1)		0.999(0.976, 1)	0.205(0.000, 0.981)	0(0, 0)
**Ewasher**	0(0, 0)	0(0, 0)	0(0, 0)		1(0.999, 1)	1(0.999, 1)	1(0.999, 1)
**RefFreeEWAS**	1(0.965, 1)	0.98 (0.392, 1)	0(0, 0.403)		0.995(0.991, 0.998)	0.994(0.991, 0.998)	0.52(0.014, 1)
**SVA**	1(0.99, 1)	1 (0.99, 1)	0.99(0.965, 1)		0.996(0.993, 0.999)	0.996(0.993, 0.998)	0.996(0.993, 0.999)
**Refactor**	1(0.965, 1)	1(1,1)	1(1,1)		0.994(0.808, 0.998)	0.042 (0, 0.695)	0(0, 0)
**RefFreeCellMix**	1(0.975, 1)	1 (1,1)	1 (1,1)		0.988(0.968, 0.996)	0.889(0.685, 0.976)	0.211(0.047, 0.525)
	**Number of Important CpGs =150**						
**Unadjusted**	0.993(0.723, 1)	1 (1, 1)	1 (1, 1)		0.999(0.965, 1)	0.204(0.000, 0.972)	0(0, 0)
**Ewasher**	0(0, 0)	0(0, 0)	0(0, 0)		1(0.999, 1)	1(0.999, 1)	1(0.999, 1)
**RefFreeEWAS**	0.993(0.973, 1)	0.98(0.318, 1)	0(0, 0.294)		0.992(0.986, 0.997)	0.992(0.986, 0.999)	0.496(0.013, 1)
**SVA**	1(0.993, 1)	1(0.993, 1)	0.993(0.970, 1)		0.992(0.988, 0.996)	0.992(0.989, 0.996)	0.993(0.988, 0.996)
**Refactor**	1(0.98, 1)	1(1,1)	1(1,1)		0.989(0.794, 0.997)	0.042 (0, 0.674)	0(0, 0)
**RefFreeCellMix**	1(0.983, 1)	1(1,1)	1(1,1)		0.975(0.929, 0.993)	0.744(0.471, 0.939)	0.098(0.022, 0.293)

Footnote**: ρ = correlation between primary covariate and latent variables. ρ = 0 corresponds to data simulated from Scenario 1, while ρ=0.7 corresponds to data simulated from Scenario 2.**
